# Lifetime Effective Dose Assessment Based on Background Outdoor Gamma Exposure in Chihuahua City, Mexico

**DOI:** 10.3390/ijerph121012324

**Published:** 2015-09-30

**Authors:** Sergio Luevano-Gurrola, Angelica Perez-Tapia, Carmelo Pinedo-Alvarez, Jorge Carrillo-Flores, Maria Elena Montero-Cabrera, Marusia Renteria-Villalobos

**Affiliations:** 1 Facultad de Zootecnia y Ecología, Universidad Autónoma de Chihuahua, Perif. Francisco R. Almada km 1, 31415 Chihuahua, Mexico; E-Mails: sergio7_mex@hotmail.com (S.L.-G.); angelicapereztapia0308@gmail.com (A.P.-T.); cpinedo@uach.mx (C.P.-A.); 2 Centro de Investigación en Materiales Avanzados, Miguel de Cervantes 120, 31136 Chihuahua, Mexico; E-Mails: carrillo@cimav.edu.mx (J.C.-F.); elena.montero@cimav.edu.mx (M.E.M.-C.)

**Keywords:** radioactivity mapping, radiation dose, health risk, kriging, Chihuahua

## Abstract

Determining ionizing radiation in a geographic area serves to assess its effects on a population’s health. The aim of this study was to evaluate the spatial distribution of the background environmental outdoor gamma dose rates in Chihuahua City. This study also estimated the annual effective dose and the lifetime cancer risks of the population of this city. To determine the outdoor gamma dose rate in air, the annual effective dose and the lifetime cancer risk, 48 sampling points were randomly selected in Chihuahua City. Outdoor gamma dose rate measurements were carried out by using a Geiger-Müller counter. Outdoor gamma dose rates ranged from 113 to 310 nGy·h^−1^. At the same sites, 48 soil samples were taken to obtain the activity concentrations of ^226^Ra, ^232^Th and ^40^K and to calculate their terrestrial gamma dose rates. Radioisotope activity concentrations were determined by gamma spectrometry. Calculated gamma dose rates ranged from 56 to 193 nGy·h^−1^. Results indicated that the lifetime effective dose of the inhabitants of Chihuahua City is on average 19.8 mSv, resulting in a lifetime cancer risk of 0.001. In addition, the mean of the activity concentrations in soil were 52, 73 and 1097 Bq·kg^−1^, for ^226^Ra, ^232^Th and ^40^K, respectively. From the analysis, the spatial distribution of ^232^Th, ^226^Ra and ^40^K is to the north, to the north-center and to the south of city, respectively. In conclusion, the natural background gamma dose received by the inhabitants of Chihuahua City is high and mainly due to the geological characteristics of the zone. From the radiological point of view, this kind of study allows us to identify the importance of manmade environments, which are often highly variable and difficult to characterize.

## 1. Introduction

In recent years, the assessment of pollution, risk and health impact caused by radioisotopes present in the environment has received great importance. Terrestrial sources produce external and internal exposure; ^232^Th and ^238^U and their descendants are the isotopes that mostly cause radiation of terrestrial origin [1]. 

Uranium and thorium are found in many crustal rocks. Uranium is highly concentrated in granite, lignite and phosphate deposits [2], whereas thorium is concentrated in monazite, granites, gabbros, gneiss and shales [3]. In soil, these radioisotopes are tightly related to the type of rocks from the zone. Consequently, radioisotopes, such as uranium, thorium, radium, radon and ^40^K, are found in building materials, contributing to radiation exposure [4]. It is important to determine the radiation dose due to both internal and external exposure received by a population. Background radiation due to radioisotopes in soils can be determined by directly measuring the absorbed dose rates in air, which is an easy way to assess the exposure levels at one site and, consequently, to predict the risk of health damage related to ionizing radiation. 

In the State of Chihuahua, about 30 uranium anomalies have been found, and most of them are located near Chihuahua City [5]. In addition, two zones, Pastorias and Marcos, located SW and NW, respectively, from Chihuahua City, could contribute radioactive minerals to the Chihuahua basin [6]. The aim of this study was to evaluate the spatial distribution of the environmental background outdoor gamma dose rates in Chihuahua City. The study also estimated lifetime cancer risks for the nearby population.

## 2. Experimental Section 

This study aimed to measure radioisotope concentrations in soil, the distribution of gamma dose rates, as well as to evaluate cancer risk resulting from exposure. To obtain the aforementioned measures, the following methods were used: (1) direct measurements of the outdoor gamma dose rate in the air; and (2) the external gamma dose rate calculated from natural radioisotopes in the soil. 

### 2.1. Study Area

**Figure 1:**
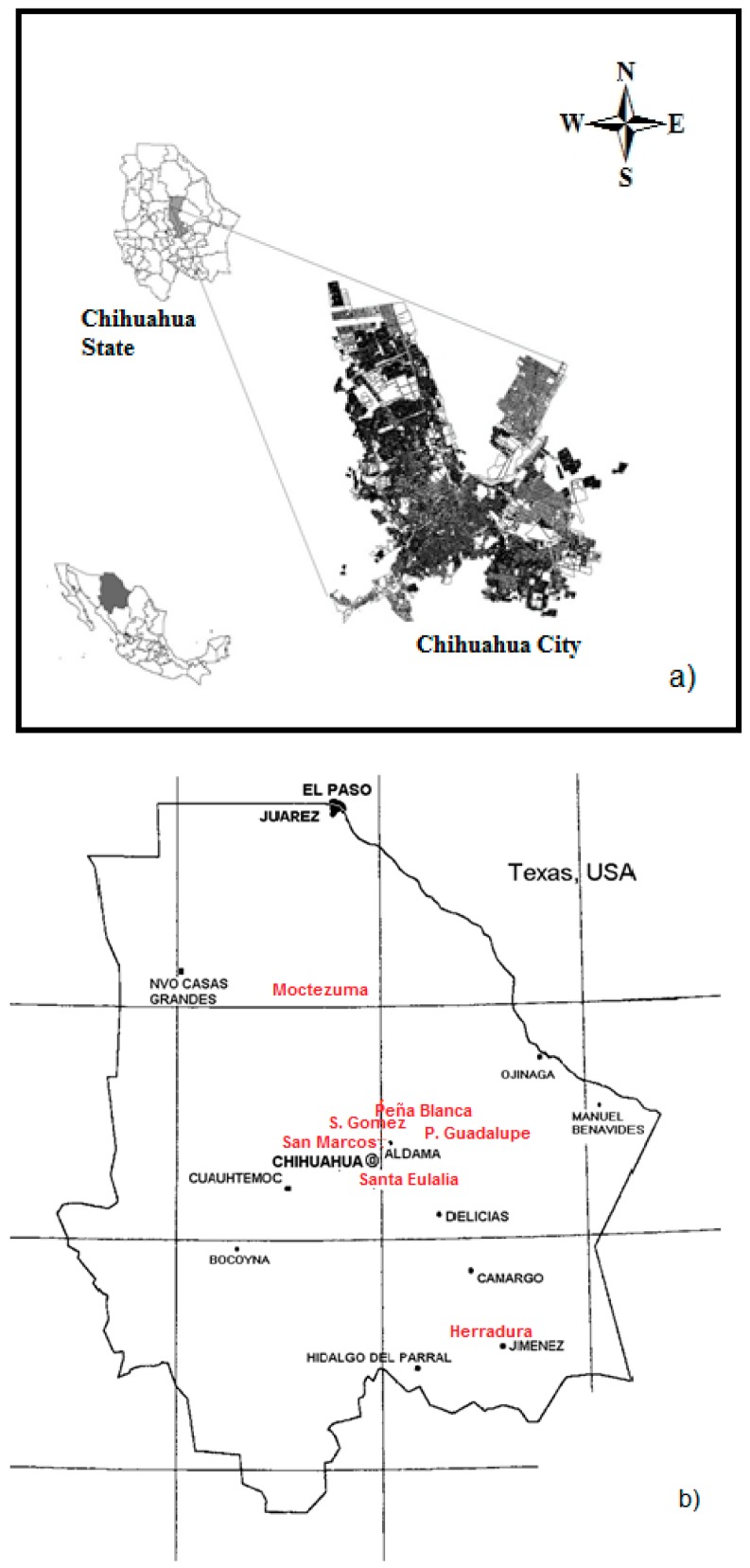

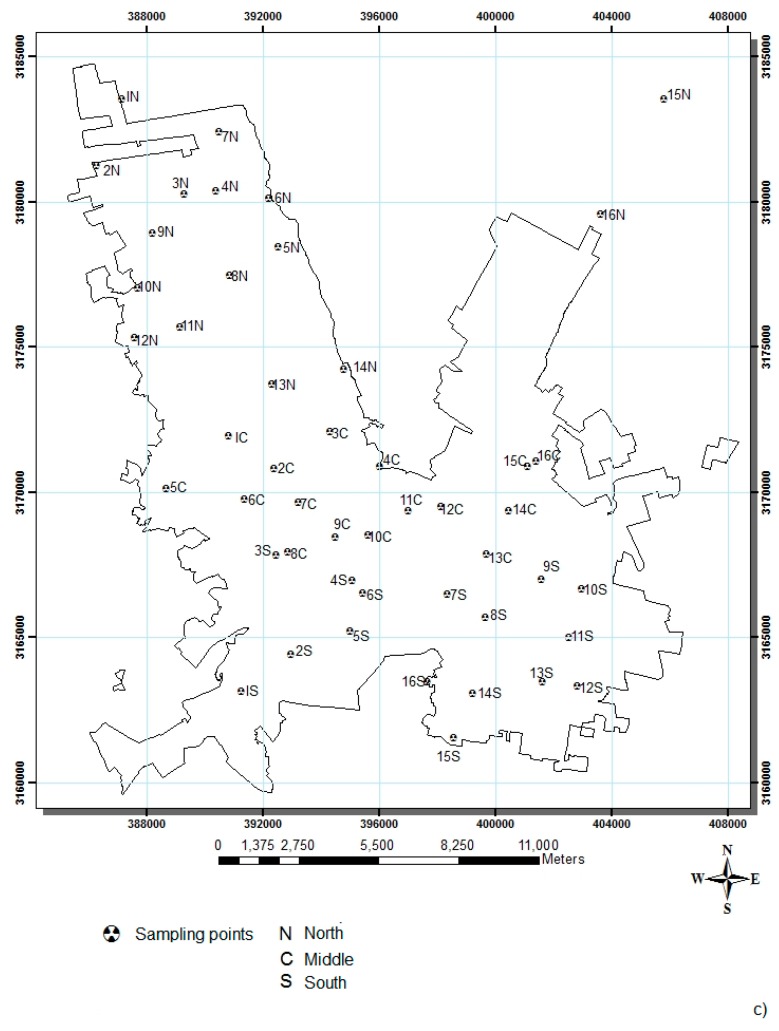
Study area: (**a**) localization; (**b**) uranium ore deposits (in red); and (**c**) sampling points.

The city of Chihuahua is located in northern Mexico (28°38′07″ N and 106°05′20″W; 1250-meter altitude), in a valley 6 km in width and 32 km in length. Its population is around 819,543 inhabitants [7]. The location of the study area is shown in Figure 1.

### 2.2. Sampling and Analysis

#### 2.2.1. Outdoor Gamma Dose Rates

To determine the outdoor gamma dose rates (*OGDR*), the city was divided into three zones of measurements; north (N), middle (C) and south (S); see Figure 1c. These zones were classified in relation to the topography of the area of study, where the middle zone is the lowest part of Chihuahua City.

In each zone, sixteen sampling points were randomly distributed. Measurements were taken in vacant lots or unpaved streets places, closer to each randomly-selected point. The location of every sampling point was obtained by a Trimble Juno GPS (Global Positioning System).

In order to evaluate the variation of *OGDR* with the measurement height, it was taken at three different heights 0.01, 0.5 and 1 m from the ground. At every sampling point, three measurements were taken. The accuracy of the measured activity concentrations was around 13%. In addition, the ArcGis 9.3 package was used to perform the spatial analysis using the geoprocessing-interpolation technique to plot the *OGDR* measures [8]. *OGDR* was taken with a portable Bicron device Model Surveyor 50 (Geiger-Müller counter). Values of *OGDR* (nGy·h^−1^) recorded were transformed to mSv·y^–1^ to obtain the annual effective dose. The annual effective dose (*AED*) was calculated by the Equation (1) described in [1]:

*AED = OGDR × DCC × OF × T* (1)

To do this, the conversion coefficient (*DCC*) from the absorbed dose in air to the effective dose received by adults (0.7 Sv·Gy^–1^), the number of hours (*T*) in a year of 365 days (8760 h) and the outdoor occupancy factor (*OF*, 0.2) were used.

#### 2.2.2. ^226^Ra, ^232^Th and ^40^K Activity Concentrations in Soil and the Terrestrial Gamma Dose Rate

Forty eight soil samples were taken in the city; to a depth around 10 cm and removing foreign bodies. Samples were ground and sieved to 2 mm in diameter. Then, samples were dried to 50 °C for 48 h. Sixty grams of the samples were placed in a Petri dish hermetically sealed and kept for one month to reach secular equilibrium. ^226^Ra, ^232^Th and ^40^K activity concentrations were determined using an HPGe coaxial detector with 20% relative efficiency, manufactured by Canberra. ^226^Ra activities were obtained by means of the 609-keV (^214^Bi) photo-peak. ^232^Th activities were determined from the 238-keV (^212^Pb) and 912-keV (^228^Ac) photo-peaks. ^40^K activity was obtained from the 1460-keV photo-peak. The detector was calibrated using RGU, RGTh and RGK (RG-set for Uranium, Thorium and Potassium; IAEA) certified reference materials and corrected for the background of the laboratory. These samples were measured during 48 h using the same geometry as the soil samples. The accuracy of the measured activity concentrations was around 10%. The absorbed gamma dose rate was calculated from activity concentrations of ^226^Ra,^232^Th and ^40^K, using the conversion factors of 0.461, 0.604 and 0.042 (nGy·h^−1^ per Bq·kg^−1^), respectively [1]. Meanwhile, the annual effective dose rate was estimated by using 0.7 Sv·Gy^−1^, 8760 h and 0.2 (the outdoor occupancy factor, *OF*), described in Equation (1). Likewise, the activity concentrations of natural radioisotopes were plotted using ArcGis 9.3.

#### 2.2.3. Assessment of Lifetime Cancer Risk

The risk estimation of cancer associated with radiation may be studied in populations living in zones with high levels of natural background exposure. The lifetime cancer risk (*LTCR*) was obtained by the Equation (2) [9]:

*LTCR = AED × LE × RFSE* (2)

*LE* is the lifetime expectancy in Chihuahua (73 years) [10], and *RFSE* is the risk factor for stochastic effects of the common population (0.05) [9].

## 3. Results and Discussion

### 3.1. Results 

Here, we are determining the spatial distribution of both the outdoor gamma dose measured and the calculated radiation dose from radioisotopes in the soil. The results of *OGDR* obtained at 1 m from the ground are shown in Table 1. 

**Figure 2:**
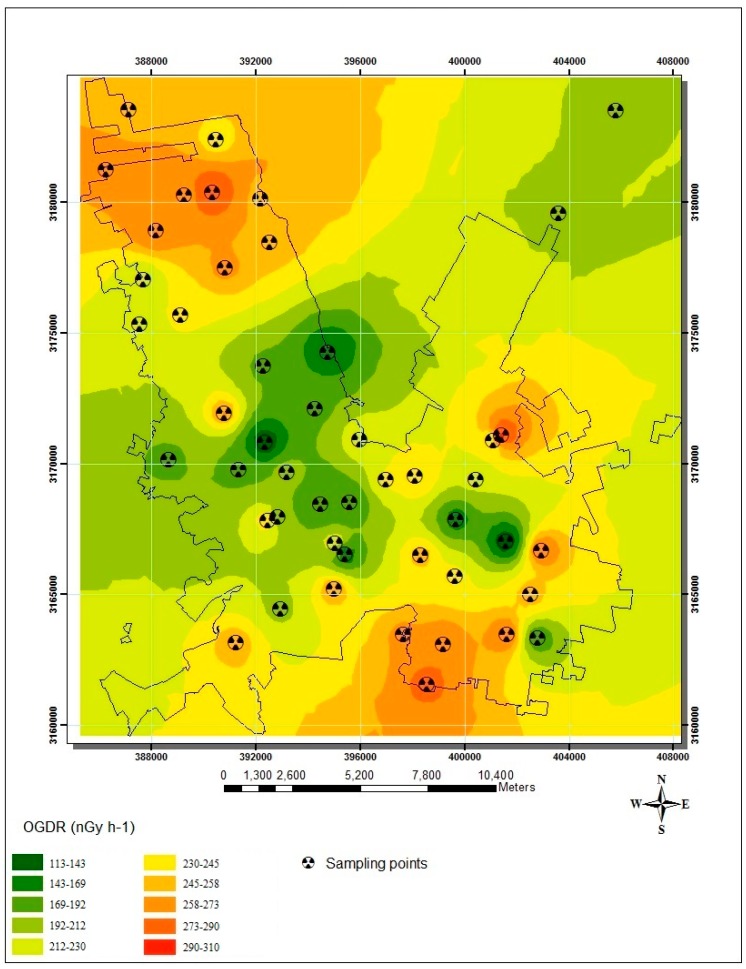
Spatial distribution of outdoor gamma dose rates in Chihuahua City.

In order to identify the statistical significance between zones (N, C and S), the results of both *OGDR* and radioisotopes in soil were analyzed using an analysis of variance (ANOVA). The ANOVA showed a difference between zones with a *p*-value of 0.97 for *OGDR* measurements. Moreover, it was found that Zone C was significantly different from Zones N and S (95% confidence, Tukey test), because this zone showed the lowest *OGDR* values. The two highest *OGDR* measurements were found in Zones C and N with values of 310 and 301 nGy·h^–1^, respectively. The spatial distribution of *OGDR* is mapped in Figure 2. Thus, the *OGDR* average in Chihuahua City was 225 ± 49 nGy·h^−1^ with a range from 113 to 310 nGy·h^−1^.

**Figure 3:**
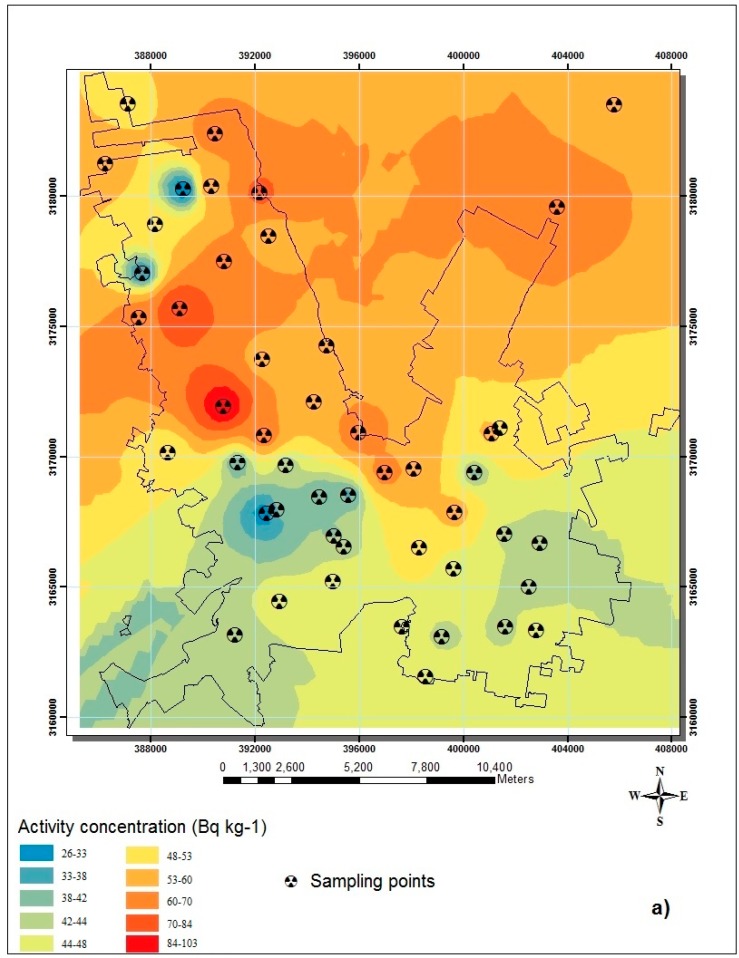

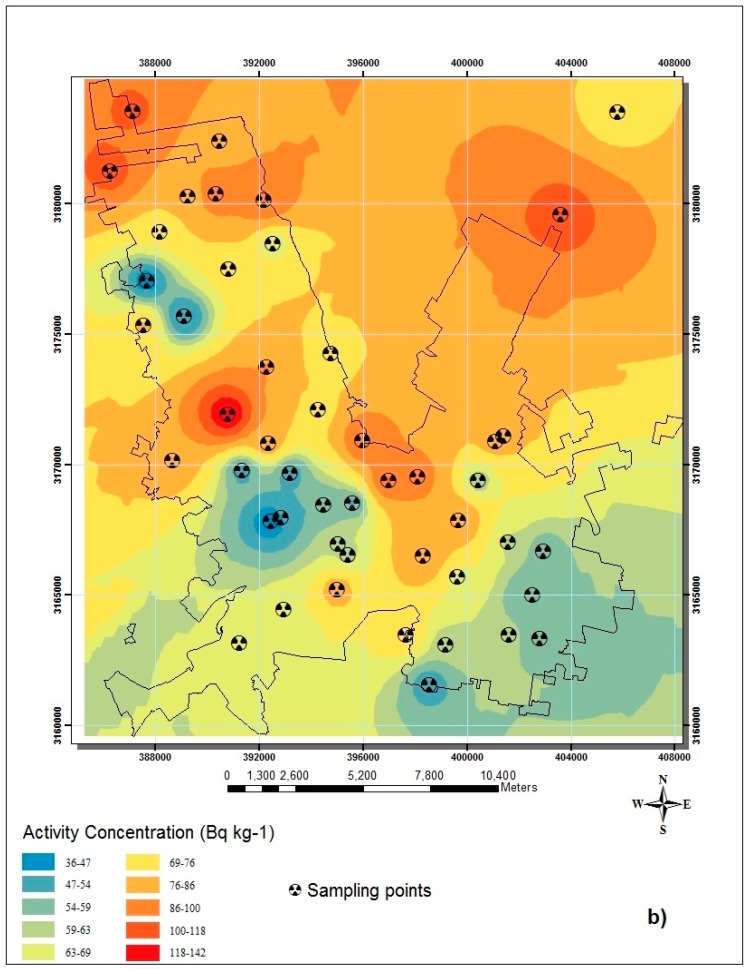

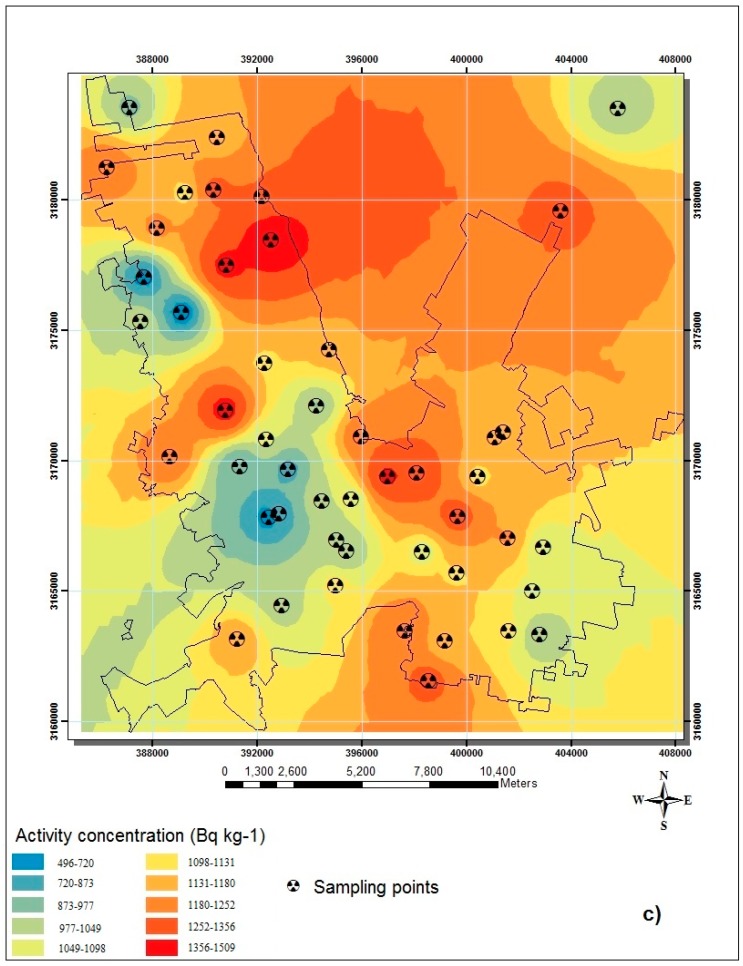
Spatial distribution of the radioisotope activity concentration found in soil: (**a**) ^226^Ra; (**b**) ^232^Th; and (**c**) ^40^K.

The results of radioisotope activity concentrations in soil and their spatial distribution are shown in Table 2 and Figure 3, respectively. The means of activity concentrations in soil were 52, 73 and 1097 Bq·kg^−1^, for ^226^Ra, ^232^Th and ^40^K (with <3% relative uncertainty), respectively. In addition, ANOVA analysis did not show a statistical significance among zones for the activity concentrations in soil of ^226^Ra (*p* = 0.005) and ^232^Th (*p* = 0.03). Otherwise, ^40^K contents were statistically differently distributed (*p* = 0.6) in the zones established. The determination of the annual effective dose (*AED*), measured in air and calculated from soil is shown Table 3.

The average of the annual effective dose for Chihuahua city was 0.28 mSv·y^−1^ (0.14–0.38). This effective dose represents the sum of contributions from terrestrial and cosmic doses. Considering a life expectancy of 72.3 years [10], the population of Chihuahua is exposed to 19.8 mSv, on average (see Table 3). 

**Table 1:** Outdoor gamma dose rates (nGy·h^–1^) measured at 1 m from the ground. C, middle.

Zone	N *	Mean ± SD ^§^	Median	25–75 Percentiles	Min–Max
N	16	235 ± 40	244	207–263	141–301
C	16	204 ± 51	197	167–235	113–310
S	16	236 ± 52	254	214–277	113–282

* n sample size; **^§^**standard deviation.

**Table 2:** Average and range of the activity concentrations (Bq·kg^−1^) of ^226^Ra, ^232^Th and ^40^K found in the soils of Chihuahua City.

Zone	^226^Ra	^232^Th	^40^K
N	58 (27–90)	79 (40–108)	1107 (496–1509)
C	54 (37–103)	77 (48–142)	1117 (794–1424)
S	44 (26–51)	63 (37–93)	1066 (521–1295)

**Table 3:** Natural gamma radiation doses, average and range, obtained from the ground in Chihuahua City.

Zone	*OGDR* (nGy·h^−1^)			Lifetime Total Effective Dose (mSv) *	Lifetime Cancer Risk *	*AED*(µSv·y^−1^)	
	From Direct Measurements	From Soil Concentrations	Remaining Dose ^§^			From Direct Measurements	From Soil Concentrations
N	235 (141–301)	121 (64–148)	79 (19–125)	21	1.04 E^−3^	288 (173–369)	148 (78–182)
C	204 (113–310)	118 (79–193)	57 (8–154)	18	0.90 E^−3^	250 (138–380)	145 (98–237)
S	236 (113–282)	103 (56–124)	101 (2–157)	21	1.04 E^−3^	289 (138–346)	126 (69–152)

^§^ Obtained by subtraction of absorbed dose contributions from soil and cosmic radiation (only directly ionizing and photo components). * Average obtained by using outdoor gamma dose rates (*OGDR*) measured to 1 m from the ground.

### 3.2. Discussion

Radioactivity concentrations in soils and rocks are the main contributors to natural background radiation [11]. The *OGDR* average calculated from the concentrations of radioisotopes in the soil of the City of Chihuahua are higher than the world’s average of 60 nGy·h^−1^. Nevertheless, the range from 56–193 nGy·h^−1^ calculated coincides with the range of 10–200 nGy·h^−1^, shown by [1]. Some countries, such as China, India and Norway, have attributed this gamma dose (200 nGy·h^−1^) mainly to the radioisotope concentration found in soils. Table 3 shows the activity concentrations of ^226^Ra, ^232^Th and ^40^K found in soil. In the soils of Chihuahua City, the activity concentrations of ^226^Ra, ^232^Th and ^40^K were higher than those reported worldwide (32, 45 and 420 Bq·kg^−1^, respectively). These higher concentrations of radioisotopes may be explained by the geology of Chihuahua’s valley. Alluvial soils are formed by matter eroded from surrounding rocks. Felsic rocks, such as rhyolites, are present in mountain ranges around Chihuahua valley. These types of rocks tend to have high contents of thorium and uranium in a range from 12 to 230 Bq·kg^−1^ [12,13]. Thus, most activity concentrations of uranium and thorium series analyzed in soil samples of Chihuahua showed values similar to those attributed to rhyolitic rocks. Furthermore, it may be observed that higher values of ^226^Ra and ^232^Th in the soil samples are present to the north of Chihuahua City. From a geological point of view, these results were expected, because in a previous study, it was found that most of the highly radioactive rocks are located to the north and west of the Chihuahua valley [14]; see Figure 1b. In addition, in this study, it was found that the highest ^226^Ra concentrations are present at two points to the west of the city. Meanwhile, high concentrations of ^232^Th were found to the northwest of it. This last finding corresponds to ^238^U and ^232^Th contents in the rocks of the San Marcos range NW of the city [15]. Similarly, Reyes-Cortés *et al.* [16] studied the radioactivity in two soil profiles of Chihuahua City. They found that ^232^Th was present in high concentrations in the soil profile located to the north of the city, whereas ^238^U and ^40^K were concentrated in the soil profile to the south. 

It is important to mention that the three radioisotopes are present in high concentration in the center of the city, Zone C (see Figure 3). In addition, an evaluation of the correlation between radioisotopes was performed. The linear correlations (R^2^) found between ^226^Ra and ^232^Th and ^232^Th and ^40^K were 0.89 and 0.71, respectively. However, a low linear correlation was found between ^226^Ra and ^40^K with R^2^ of 0.44. These high activity concentrations may be due to the topography of the region. Zone C is placed at the lowest level of the Chihuahua valley, retaining eroded material from surrounding mountain ranges. In addition, in the San Marcos area, located NW of Chihuahua City, there are two uranium mineral outcrops [17]. Previous studies have demonstrated that this area shows higher radioactivity levels in surface water, sediments and biota [18]. Likewise, the authors have found that there is mobility of the uranium by surface and ground water from the San Marcos area to the low levels of the Chihuahua valley [19]. Thereby, water from San Marcos may transport uranium ions in solution, which could be trapped in clays and/or organic material of the soil of Zone C, since this alluvial soil has been forming. Furthermore, high concentrations of ^40^K were found in the south of the study area. This may be due to, in addition that described above, some agricultural lands that in the past years were located to the south of the city. These agricultural activities involved the use of fertilizers that possibly had high potassium concentrations [20,21]. 

**Figure 4:**
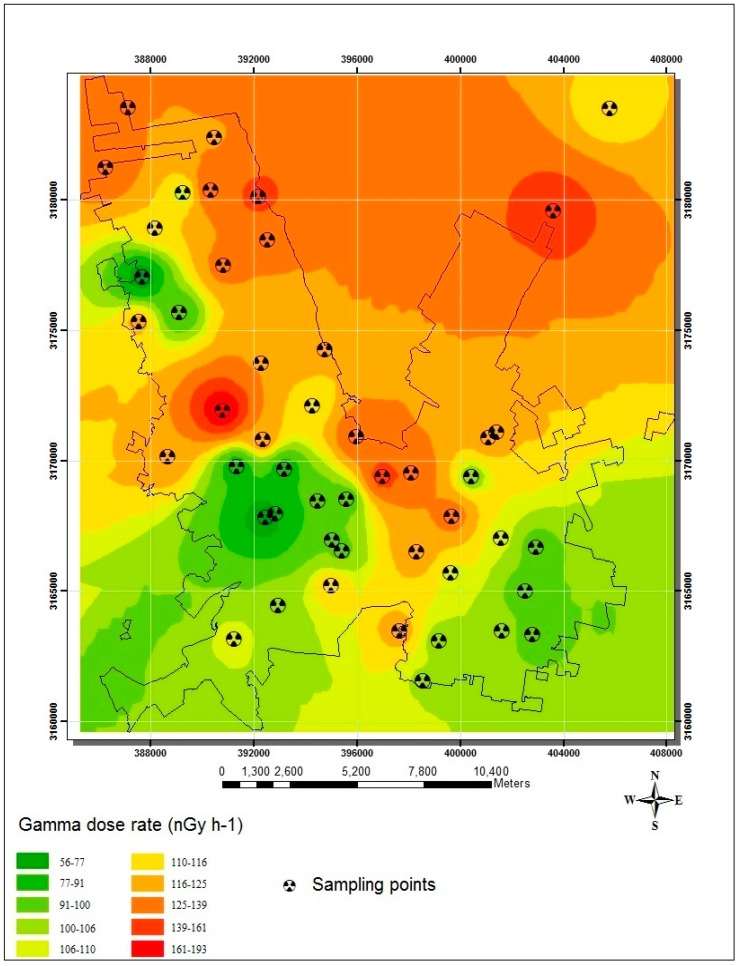
Spatial distribution of absorbed gamma dose rates calculated from the soil activity concentrations in Chihuahua City.

The distribution of *OGDR* calculated from radioisotope concentrations in soil is shown in Figure 4. 

On the other hand, the state of Chihuahua is the largest in Mexico. Consequently, urbanization is increasing across the territory that is available. Nevertheless, the downtown of this city, which corresponds to Zone C, presents a larger number of covered streets. In this zone, most of the *OGDR* measurements were taken within building lots; however, the distance between street cover and sampling points did not exceed 12 m. This may explain the lowest *OGDR* values in this zone, by gamma attenuation. This can be observed from the fact that the *OGDR* median value is lower than the *OGDR* average value in Zone C (Table 1). Conversely, in Zones N and S, there are several places with vacant areas and unpaved streets, allowing better *OGDR* measurements without contributions from others factors. 

From the results shown in Table 3, a great difference between the *OGDR* measured directly at 1 m and the *OGDR* calculated from radioisotopes in soil may be observed. However, in the estimation of the absorbed dose rate from direct measurement, there are several environmental factors that have an important role at the ground level. The main contributors are terrestrial radioisotopes, cosmic radiation and environmental elements, such as building materials [1]. 

The absorbed dose contribution by cosmic rays has been widely studied around the world, the altitude and latitude being the main factors of the absorbed dose variation. The UNSCEAR (United Nations Scientific Committee on the Effects of Atomic Radiation) 2000 report showed an estimated absorbed dose contribution of 30 nGy·h^−1^ for latitudes below 30° [1]; muons the being the main contributor with approximately 80% of the absorbed dose at the ground level. Chihuahua City is located at a latitude of 28°38′07″ and an altitude of 1250 m where, assuming an exponential increase, the altitude factor would be 1.36. The absorbed dose contribution by only the ionizing and photon components would be 41 nGy·h^−1^ (30 nGy·h^−1^ multiplied by 1.36). This information was used to calculate the estimated remaining absorbed dose rate, subtracting the dose contributions by both terrestrial radioisotopes and cosmic radiation from direct *OGDR* measurements; see Table 3. On average, the *OGDR* values obtained by direct measures at 1 meter could be composed of around 18% cosmic radiation and 50% calculated radiation dose from the radioisotopes in soil. The remaining 32% can be attributed to the sum of factors added to the contribution of gamma emission from building materials. From this analysis, there were four sampling points (14N, 2C, 13C and 9S) with closer *OGDR* values between direct measurements and those calculated from soil; these sampling points showed the lowest *OGDR* values measured at 1 m from the ground. The results indicated a clear variability of the absorbed dose rate among zones, where Zone C showed the lowest average value (median value of 50 nGy·h^–1^). In this zone, the *OGDR* values from terrestrial radioisotopes are similar to the results found in Zones N and S, even slightly higher. Therefore, that variability may corroborate the gamma attenuation by street cover and buildings, which may be acting as a shield. However, in Zones N and S, the shielding factor is less present. These zones showed an average of remaining absorbed dose of 79 and 101 nGy·h^−1^, respectively. External exposures outdoors are widely related to radioisotope concentrations in the soil. Nevertheless, there are several conditions that may induce underestimation of the absorbed dose rate of these direct measurements. In order to understand the variability of absorbed doses and to improve the findings, it is necessary to highlight some aspects. Some of these may be from the methodological procedure, such as the container’s material for measurements and the kind of sealing used (it may present underestimation of radioisotope concentrations by radon escape during the period for reaching secular equilibrium), the representative sample of soil at 10 cm (because the radiation comes from around 50 cm layer in the soil), the inherent background of the dose monitor and the calibration of the monitor. Others aspects can be the influence of moisture in the soil, the deposition of radon progeny by rain and the absorbed dose contribution by neutrons in the cosmic radiation, among others. However, the information obtained in this research gives insight into further detailed studies.

Finally, it is important to estimate the exposure produced at environmental levels and the annual effective dose resulting for the population. The lifetime cancer risk estimate is a useful tool to obtain a better understanding of the health effects produced by a low radiation dose. The lifetime effective dose for inhabitants of Chihuahua City is on average 19.8 mSv, resulting in a lifetime cancer risk of 0.001. These results are low for producing some health effects, if compared to those reported in zones which that have been exposed to elevated levels of radiation by nuclear accidents [1]. However, from the natural background radiation, the lifetime total effective dose received by the population in Chihuahua is higher than the results reported by some authors [22,23] or the same order [24]. 

Currently, more efforts are being made to obtain more information on the relationship of low levels of gamma exposure and indoor radon with the induction of cancer [1]. This is because the continued exposure to low gamma radiation may potentiate the risk of some kinds of cancer. Unfortunately, in this study, it may not be possible to evaluate this correlation. Moreover, it is necessary to measure the indoor external exposure produced by building material, as well as indoor radon (the main source of the dose in most countries), which, in addition to outdoor external exposure, give a better understanding of the effective dose received by populations. Nevertheless, the importance of this kind of study allows us to establish the baseline for the radiation dose to which the inhabitants of the city of Chihuahua are exposed.

## 4. Conclusions 

In this study, it was found that the activity concentrations of ^226^Ra, ^232^Th and ^40^K in soil samples are above the worldwide average. In addition, the variability in the spatial distribution of radioisotopes was obtained. The distribution of ^232^Th, ^226^Ra and ^40^K is to the north, to the north-center and to the south of the city, respectively. The characteristic rocks, rhyolites, contribute to the high contents of uranium and thorium observed in the alluvial soil. The outdoor gamma dose, from direct measurements and obtained from the radioisotope concentration in the soil, is higher in comparison to the natural background average reported by the UNSCEAR Report 2000. The high values of *OGDR* measured at 1 m from the ground may be attributed to the sum of gamma doses from building materials and cosmic rays, despite the high terrestrial concentrations. In conclusion, the natural background gamma dose received by inhabitants of Chihuahua City is high and mainly due to the geological characteristics of the zone. From the radiological point of view, this kind of study allows us to identify the importance of manmade environments, which are often highly variable and difficult to characterize.
